# Surface proteins of Shiga toxin-producing *Escherichia coli* mediate association with milk fat globules in raw milk

**DOI:** 10.3389/fmicb.2023.1156374

**Published:** 2023-06-23

**Authors:** Arthur Bagel, Marion Bouvier-Crozier, Mélissa Canizares, Badis Hamadou, Louise Courcol, Christelle Lopez, Valérie Michel, Thomas Douellou, Delphine Sergentet

**Affiliations:** ^1^Bacterial Opportunistic Pathogens and Environment Research Group, UMR 5557 Ecologie Microbienne Lyon, CNRS, Université de Lyon, VetAgro Sup, INRAE, Marcy-l’Etoile, France; ^2^Laboratoire d’Etudes des Microorganismes Alimentaires Pathogènes—French National Reference Laboratory for Escherichia coli Including Shiga Toxin-Producing E. coli (NRL-STEC), Université de Lyon, VetAgro Sup—Campus Vétérinaire, Marcy-l’Etoile, France; ^3^INRAE, UR BIA, Nantes, France; ^4^Actalia, La Roche-sur-Foron, France

**Keywords:** bacterial anti-adhesion, MFGMPs, STEC, flagellin, food

## Abstract

**Introduction:**

By adhering to host cells and colonizing tissues, bacterial pathogens can successfully establish infection. Adhesion is considered the first step of the infection process and bacterial adhesion to anti-adhesive compounds is now seen as a promising strategy to prevent infectious diseases. Among the natural sources of anti-adhesive molecules, the membrane of milk fat globules (MFGs) is of interest because of its compositional diversity of proteins and glycoconjugates. However, few studies have focused on the bacterial molecules involved in MFG- mediated inhibition of bacterial adhesion to enterocytes.

**Methods:**

We used three pathogenic Shiga toxin-producing Escherichia coli (STEC) strains (O26:H11 str. 21765, O157:H7 str. EDL933, and O103:H3 str. PMK5) as models to evaluate whether STEC surface proteins are involved in the affinity of STEC for MFG membrane proteins (MFGMPs). The affinity of STEC for MFGMPs was assessed both indirectly by a natural raw milk creaming test and directly by an adhesion test. Mass spectrometry was used to identify enriched STEC proteins within the protein fraction of MFGMs. Bacterial mutants were constructed and their affinity to MFGs were measured to confirm the role of the identified proteins.

**Results:**

We found that free STEC surface proteins inhibit the concentration of the pathogen in the MFG-enriched cream in a strain-dependent manner. Moreover, the OmpA and FliC proteins were identified within the protein fraction of MFGMs. Our results suggest that FliC protein participates in STEC adhesion to MFGMPs but other STEC molecules may also participate.

**Discussion:**

For the first time, this study highlighted, the involvement of STEC surface proteins in the affinity for MFGs. The mechanism of STEC-MFG association is still not fully understood but our results confirm the existence of receptor/ligand type interactions between the bacteria and MFGs. Further studies are needed to identify and specify the molecules involved in this interaction. These studies should consider the likely involvement of several factors, including adhesion molecules, and the diversity of each STEC strain.

## 1. Introduction

Bacterial adhesion is the first step of infection. To effectively colonize hosts and cause symptoms, pathogenic bacteria use adhesive strategies to attach to host cells (Pizarro-Cerdá and Cossart, 2006). Adhesion is necessary so that bacteria are not cleared by the host’s natural defense mechanisms. The bacterial adhesion process consists of a non-specific phase, involving physicochemical interactions, and a specific phase involving molecular factors exposed on both host and bacterial cell surfaces (Ofek et al., 2013). Inhibiting the bacterial adhesion step has been reported to be a key strategy for infection control (Asadi et al., 2019).

Prevention of bacterial adhesion is now considered a promising strategy to reduce the occurrence of infectious diseases. Several specific approaches have been considered, including inhibition of adhesion by membrane receptor analogs (Leonard et al., 2019). Several natural molecules contained in food, especially milk components (Horemans et al., 2012; Douëllou et al., 2017b; Bagel and Sergentet, 2022), could act as effective inhibitors of pathogen adhesion (Ofek et al., 2003, 2013; Sharon, 2006; Kahane and Ofek, 2012; Orth and Krachler, 2013). Many studies have shown that the association of bacteria with raw milk fat globules (MFGs) could prevent the adhesion of several enteropathogens to enterocytes through mimetic receptors (Douëllou et al., 2017b; Bagel and Sergentet, 2022). Our objective was now to better understand the underlying mechanisms in order to develop tools to prevent food contamination and develop strategies to treat infections.

MFGs are present in raw milk as triglyceride (TAG) microdroplets surrounded by a complex biological membrane, called the milk fat globule membrane (MFGM), organized as a trilayer of polar lipids embedding proteins which ensure their integrity and individuality (Lopez, 2020). The MFGM is mainly composed of lipids (64 to 71.8%) and proteins (22.3 to 28%) (Ortega-Anaya et al., 2022), of which approximately 10% are glycosylated (Ross et al., 2016). The MFGM protein fraction shows similarities to that of intestinal cells (Jiménez-Flores and Brisson, 2008).

Among enteric bacteria responsible for foodborne infections, some strains of *Escherichia coli*, the Shiga-toxin producing *E. coli* (STEC), are responsible for large-scale epidemics. STEC-related outbreaks have been described in several countries, making this pathogen an international public health issue (FAO and WHO, 2019). STEC are frequently associated with severe forms of infection such as hemorrhagic colitis (HC). In very severe cases, STEC may cause systemic complications in the form of hemolytic uremic syndrome (HUS), cerebral failure over several years, and in some cases, death of the patient. Children and the elderly are most likely to develop HUS, which is the leading cause of renal failure in children. In 2010, there were more than 1.2 million cases of foodborne STEC infections, which caused 128 deaths and nearly 13,000 Disability-Adjusted Life Years (DALYs) (WHO, 2015).

Ruminants are the primary reservoir of STEC (Farrokh et al., 2013). Despite the efforts and resources expended by farms to control this pathogen, human infection is most often linked to ingestion of contaminated food and water such as undercooked ground meat, raw milk cheese, or raw plant material. Nevertheless, raw milk products are only a minor source of human enteric infection (EFSA and ECDC, 2019, 2021a,b). Interestingly, STEC prevalence data in dairy matrices and STEC outbreaks do not fit overall foodborne-related outbreak figures (EFSA and ECDC, 2021b). Douellou et al. hypothesized that this phenomenon might be related to an association between STEC and MFGs, which may inhibit STEC adhesion to enterocytes (Douëllou et al., 2018). Bacterial affinity for MFGs and more specifically for the MFGM is accepted for various bacterial species, particularly for lactic acid and propionic bacteria (Brisson et al., 2010; Douëllou et al., 2017b; Gomand et al., 2018; Guerin et al., 2018).

To investigate this hypothesis, our team used the natural creaming of raw milk as a model. Natural creaming can concentrate bacteria and bacterial levels in cream can be up to 500 times higher than in milk (Anderson, 1909; Lamson, 1918). During creaming, MFGs spontaneously rise to the top surface because they have a lower density than the aqueous phase of milk. This forms a MFG-concentrated layer, called the cream (Mulder and Walstra, 1974). Previous studies from our team showed that 15 STEC strains, belonging to three key serotypes responsible for infection, were recovered mainly from the cream layer after natural creaming (Douëllou et al., 2018; Bagel et al., 2022a).

Furthermore, we showed that the concentration of STEC in the cream layer was dependent on the fat level of the milk and the strain of bacteria (Bagel et al., 2022a). Microscopic observations showed that *E. coli* cells were localized near MFGs, suggesting that MFGs could have a pivotal role in the phenomenon of bacterial concentration (Bagel et al., 2022a). Such data suggested that STEC could bind MFGs during natural creaming, probably through specific interactions between STEC surface proteins and sugar epitopes attached to glycoproteins and glycolipids anchored in the MFGM. The type of surface proteins exposed by different STEC strains and the glycosylated moieties recognized at the MFGM could explain, in part, the strain-dependent difference in affinity for the cream layer.

Few data exist for *E. coli* and most published studies focus on the MFGM molecules recognized by bacteria (Bagel and Sergentet, 2022). Very little data has been published on the bacterial molecules involved in this association. We hypothesized that the concentration of STEC in the cream layer during the natural creaming of raw milk is due to specific interactions between STEC surface proteins and MFGM proteins. The aims of this study were: (1) to evaluate the involvement of STEC surface proteins in the phenomenon of STEC concentration in raw milk cream and (2) to identify which STEC molecules are involved in this phenomenon.

We used three highly pathogenic STEC strains of different serotypes (O157:H7, O26:H11, and O103:H2) as model strains to investigate the role of STEC surface proteins in the affinity of STEC for MFGM. For this purpose, we performed competitive creaming assays between STEC and free STEC surface proteins and identified STEC surface proteins associated with the MFGM by proteomic analysis. The role of the outer membrane protein A (OmpA) and the flagellin (FliC) in the STEC-MFGM association was assessed by natural creaming and plate adhesion assay.

## 2. Materials and methods

### 2.1. Bacterial strains, culture conditions, and plasmids

Three pathogenic strains of Shiga Toxin-producing *Escherichia coli* (STEC) (*E. coli* with the *eae* and *stx* genes) isolated from humans were selected from the collection of the French National Reference Laboratory (LMAP, VetAgro Sup, Marcy l’Etoile, France) (Table 1). Bacteria were stored at −80°C in Brain Heart Infusion (BHI, BioMérieux, Marcy-L’Etoile, France) supplemented with 15% glycerol. Bacteria were plated from glycerin-BHI frozen stock (−80°C) on LB agar plates (Oxoïd) and incubated at 37°C or 30°C for 16–18 h. The day before each experiment, one colony was picked from the plate and cultured overnight at 37°C or 30°C in BHI or BHI supplemented with 100 μg/mL of ampicillin to maintain the plasmid pKD46 necessary to generate the mutants (Table 1).

**Table 1:** Plasmids and *E. coli* strains used in the experiments.

Plasmid or strain	Description	Source or reference
pKD46	λ Red-expression under control of an arabinose-inducible promoter, temperature-sensitive, Amp^R^	Datsenko and Wanner (2000)
pKD4	Kan resistance gene cassette-containing plasmid; Amp^R^ Kan^R^	Datsenko and Wanner (2000)
DH5alpha	Non-pathogenic *E. coli* strain used for plasmid multiplication	Datsenko and Wanner (2000)
BW25141	Non-pathogenic *E. coli* strain used for plasmid multiplication	Datsenko and Wanner (2000)
EDL933 WT	Pathogenic STEC O157:H7 strain (eae+, stx1+, stx2+). ATCC 43895	Perna et al. (2001)
EDL933/pKD46	EDL933-containing pKD46 used for λ Red recombination	This study
EDL933Δ*ompA*	EDL933 with *ompA* gene deleted; Kan^R^	This study
EDL933Δ*fliC*	EDL933 with *fliC* (flagellin) gene deleted; Kan^R^	This study
21765	Pathogenic STEC O26:H11 strain (eae^+^, stx1^−^, stx2^+^)	Galia et al. (2015)
21765/pKD46	21765-containing pKD46 used for λ Red recombination	This study
21765Δ*ompA*	21765 with *ompA* gene deleted; Kan^R^	This study
21765Δ*fliC*	21765 with *fliC* (flagellin) gene deleted; Kan^R^	This study
PMK5	Pathogenic STEC O103:H2 strain (eae^+^, stx1^+^,stx2^−^)	Mariani-Kurkdjian et al. (1993)
PMK5/pKD46	PMK5-containing pKD46 used for λ Red recombination	This study
PMK5Δ*ompA*	PMK5 with *ompA* gene deleted; Kan^R^	This study
PMK5Δ*fliC*	PMK5 with *fliC* (flagellin) gene deleted; Kan^R^	This study

The plasmid pKD4 was used as a DNA template for the kanamycin-resistant cassette during PCR amplification of the homologous fragmentation flanked by the upstream and downstream regions of the gene to be mutated (Table 1). Plasmid pKD46 encodes the Red genes (*γ*, *β*, exo) under the control of an arabinose-regulated promoter. It is also temperature-sensitive for easy curing at high temperatures (37–42°C) and expresses ampicillin resistance as a selection marker. The pKD4 and pKD46 plasmids were isolated from *E. coli* strains BW25141 and DH5alpha, respectively, from a 10 mL overnight culture in BHI supplemented with 100 μg/mL ampicillin and incubated at 30°C. Plasmids were isolated with ChargeSwitch – Pro Plasmid Miniprep Kit (Thermo Fisher Scientific, Invitrogen, Waltham, United States). For experiments related to mutant characterization, mutated STEC strains were grown on antibiotic-free medium.

### 2.2. STEC surface protein extraction

The extraction protocol for STEC surface proteins was performed as previously described (Bagel et al., 2022b). Briefly, bacterial stationary cultures (BHI, 37°C) were centrifuged at 5,000 g for 5 min, washed twice in PBS (vol/vol), re-suspended in 100 μL of PBS, heated at 60°C for 30 min, and centrifuged at 5,000 g for 5 min. Surface proteins from the supernatants were filtered through a 0.22 μm cellulose acetate microfiltration tube (Dutscher) at 13,000 g for 3 min to eliminate any remaining bacterial cells. Surface protein extracts were used immediately for downstream applications. However, this method could be biased because some proteins and protein parts could be lost in the cellulose.

### 2.3. Natural creaming assay of STEC in competition with STEC surface proteins

Bovine raw milks were purchased either from the local market or directly from farms (Lyon and surrounding towns, France), and were stored at 4°C before use on the day of purchase. Raw bovine milk is classically characterized by a pH = 6.8 and a fat content of 40 g/L (Boutonnier, 2008; Snappe et al., 2010). The milks were purchased over a short period of time. This means that they may come from different animals, different milking and could have small differences microbiological compositions.

#### 2.3.1. Effect of STEC surface protein concentration on STEC concentration factor in the cream layer

To assess the ability of STEC surface proteins to inhibit STEC concentration in the cream layer, 10 mL of raw milk were supplemented with a 10-fold range of concentrations of STEC surface proteins (the same strain), up to a final concentration in the product of an order of magnitude of 10^2^ μg/mL. Supplemented raw milk was stored for 2 h at 4°C without agitation to allow the possible binding of STEC surface proteins to MFGM. Then, supplemented raw milk was contaminated at 6 log_10_ CFU/mL^−^ with the same strain used as a protein supplementation source according to the OD_600_/CFU.mL^−1^ relation. This concentration was chosen because none of the strains saturated under this condition according to our previous study (Bagel et al., 2022a). We collected 100 μL from each *E. coli*-contaminated milk, then serially diluted the aliquots in PBS buffer (pH 7.3) (Oxoïd, Thermo Fisher Scientific) and immediately plated them in duplicate on Luria Bertani (LB) agar plates (Oxoïd, Thermo Fisher Scientific). Plates were incubated for 18–24 h at 37°C and colonies were counted. PCR tests as well as agglutination tests have been implemented in order to correctly count STEC. Supplemented and inoculated raw milk was placed at 4°C for 16–24 h to allow natural cream separation (creaming). We collected 100 μL of the cream layer in each tube and STEC were quantitated as described above for raw milk.

#### 2.3.2. Evaluation of the specificity of STEC protein extracts for STEC inhibition in the cream layer (crossing assay)

Strain-specific inhibition was evaluated for each strain by crossing the origin of the protein extracts as a supplementation source. For this purpose, 10 mL of raw milk were supplemented with STEC surface proteins from each strain to a final concentration of 0.20 μg/mL as described previously. After 2 h at 4°C, supplemented-raw milk was inoculated at 6 log_10_ CFU/mL with each STEC strain. The experiments were then completed as previously described. The concentration factor (CF) of STEC in the cream layer was calculated as the following formula:


$$\begin{array}{l} \mathrm{CF}=\log_{10}\left(\mathrm{Concentration of STEC in}\;\mathrm{Raw}\;\mathrm{Milk}\right)\\ \;\;\;\;\;\;\;\;\;-\log_{10}\left(\mathrm{Concentration of STEC in Cream}\right) \end{array}$$

If CF is equal to 1, there a bacteria homogeneity in the cream and in the initial sample.

If CF is greater than 1, it means that the bacteria are concentrated in the cream.

If CF is less than 1, the bacteria have been excluded from the cream.

### 2.4. Identification of STEC proteins associated with the MFGM

#### 2.4.1. Extraction of STEC proteins associated with the MFGM

For each strain, 10 mL of bovine raw milk was supplemented with 100 μL of STEC protein extract containing 1 mg of protein material, gently homogenized by turning over, and then natural creaming was performed at 4°C for 16 h–18 h. As a control, 100 μL of PBS was used in place of the STEC surface proteins. The next day, 600 μL of the cream layer were suspended in 1.4 mL of 50% w/w sucrose solution (phosphate buffer, pH = 6.8) and heated at 40°C for 5 min. MFGs were washed as described in (Patton and Huston, 1986) to eliminate STEC proteins that did not adhere to MFGs. Each heated cream sample was placed at the bottom of a 15 mL conical centrifuge tube (Falcon, Corning Life Sciences, Hazebrouck, France) filled with 8 mL of a 5% w/w sucrose solution and centrifuged for 20 min at 1600 g (Sorvall ST 16R centrifuge, Thermo Fisher Scientific) as previously reported (Obeid et al., 2019). MFGM proteins and STEC proteins associated with the MFGM were extracted as described in Cebo et al. (2010) with some modifications. Washed MFGs were suspended in 200 μL of denaturing buffer (63 mM Tris–HCl, 2% SDS, pH 6.8) supplemented with EDTA-free Protease Inhibitor Cocktail (cOmplete Mini, Merck) at the concentration recommended by the manufacturer. Samples were incubated for 1 h at 20°C with occasional vortexing and then centrifuged for 10 min at 10,000 g. The aqueous phase containing extracted proteins was recovered and quantified with the Rapid Gold BCA Protein Assay Kit (Pierce^™^ Thermo Fisher Scientific). The samples were stored at −20°C until proteomic analyses were performed.

#### 2.4.2. Label-free relative quantification of STEC proteins associated with the MFGM

Approximately 40 μg of supplied proteins were prepared following instructions from the easyPep kit from Thermo Fisher Scientific. Samples were reduced and alkylated for 10 min at 95°C and then digested with LysC/Trypsin at a 1:10 ratio for 3 h at 37°C. Samples were then purified on the easyPep kit spin columns, dried, and taken up in 50 μL 0.1% formic acid. The samples were then assayed by a fluorometric assay.

The same quantity of each sample was analyzed on a high-resolution orbitrap mass spectrometer in TOP20 HCD mode. MS data were reprocessed with Proteome Discoverer 2.5 software (Thermo Fisher Scientific) with the Sequest HT search engine against (i) the total UniProt *E. coli* database (862,106 entries; October 2021), (ii) UniProt *Bos Taurus* database (37,539 entries; November 2021), and (iii) the proteomic sequences of the three STEC strains obtained from genomic sequences via NCBI Prokaryotic Genome Annotation Pipeline (PGAP) (EDL933: 5,425 entries; 21765: 5,361 entries; PMK5: 1,994 entries) as well as the addition of a contaminant database, filtered at a false positive rate of 1%. Quantification ratios between conditions were calculated by pairwise comparison and t-test directly in Proteome Discoverer software. The data presented in the study are deposited in the MassIVE repository, accession number MSV000090906.

Further analyses were performed in R software (R Core Team, 2021) and the Venn diagrams were realized with the ‘ggvenn’ package (Yan, 2022). To identify the proteins associated with the STEC extract, the proteins identified by MS/MS were filtered according to two conditions: (i) proteins were annotated to the *Escherichia coli* species and (ii) proteins were absent from the three biological replicates of samples not supplemented with surface proteins (PBS). Strain-specific differentially expressed proteins (DEPs) were extracted based on the values of the log2 abundance ratio <−1 or >1 (according to the order of the ratio) and a value of *p* <0.05, against the other two strains. For poorly annotated proteins, a homologous protein (>90%) was used to predict function and subcellular location.

### 2.5. Bacterial mutant construction

The λ red system, adapted from Datsenko and Wanner (2000), was used to make isogenic knockouts (KOs). Gene KOs were replaced with the kanamycin (Kan) resistance cassette from pkD4 by homologous recombination.

#### 2.5.1. Overlapping DNA amplicons

The Kan resistance cassette was amplified by overlap PCR from the template plasmid pKD4 and hybrid primers. Primers were synthesized (Thermo Fisher Scientific) with 20 nucleotides of pKD4 priming sites and with 50 nucleotides from each side of the target genes (Supplementary Table S1). Extension of overlapping DNA amplicons was performed in a CFX96 PCR system (BioRad, Marnes-la-Coquette, France) with the following program: an initial denaturation of 30 s at 98°C; then 35 cycles of 10 s at 98°C, 30 s at 56°C, and 120 s at 72°C. The PCR products were purified with the Wizard SV Gel and PCR Clean-Up System (Promega), digested with DpnI (Thermo Fisher Scientific), re-purified, and finally suspended in sterile DNAse-free water.

#### 2.5.2. Bacterial transformation

Target strains were transformed with pKD46 by electroporation. On the day of each experiment, overnight cultures were diluted (1:1,000) in 40 mL of LB and cultured at 37°C until reaching an OD_600_ of ≈0.5. Bacterial cultures were centrifuged for 5 min at 5,000 g and washed twice with ice water, decreasing the volume by half between each wash. Finally, washed bacterial cell pellets were re-suspended in 400 μL of ice water supplemented with 15% glycerol. Electroporation was performed using the Gene Pulser system (BioRad) with the following parameters: 2.5 kV; 25 μF; and a 0.20 cm cuvette (BioRad) filled with 20 μL of competent bacterial cells and 1–5 μL purified pKD46 (≈10–100 ng). One milliliter of warmed LB (37°C) was instantly added to the shocked cells and transformants were recovered 1–2 h at 37°C. One-half of the recovery culture (0.5 mL) was centrifuged for 5 min at 5,000 g, re-suspended in 0.1 mL of LB, and spread onto LB agar supplemented with 100 μg/mL of ampicillin (Thermo Fisher Scientific) to select transformants. Transformants were checked by PCR and stocked as previously described at −80°C in BHI-glycerol.

For isogenic KO, transformants carrying pkD46 were made electrocompetent and transformed as previously described except that fresh cultures were realized in LB supplemented with 100 μg/mL of ampicillin and 1 mM L-arabinose and cultured at 30°C with gentle agitation. For electroporation, 1–10 μL of overlapping PCR amplicon containing 10–100 ng of DNA material was used. Bacterial cells that had undergone homologous recombination were recovered on LB agar supplemented with 50 μg/mL of kanamycin (Sigma Aldrich). Insertion of the Kan resistant cassette was checked by two PCRs upstream and downstream of the target gene. The loss of pKD46 was confirmed by an ampicillin sensitivity test. Enzymatic digestion and amplicon sequencing were performed to confirm disruption of target genes.

### 2.6. Impact of STEC gene deletions on saturation of the cream layer of raw milk by natural creaming

Natural creaming assays were performed as previously described in Bagel et al. (2022a) with some modifications. Briefly, for each *E. coli* strain, an adequate volume of overnight bacterial suspensions, containing approximately 3 × 10^9^ CFU according to the OD_600_/CFU.mL^−1^ relation, was centrifuged for 5 min at 5,000 g using a Sorvall ST 16R centrifuge (Thermo Fisher Scientific). The pellets were suspended in 10 mL of bovine raw milk to obtain a final concentration of approximately 8.5 log_10_ CFU/mL. Three different milks were used and contaminated with independent STEC cultures. A series of 10-fold dilutions was performed in raw milk to obtain four 9 mL samples contaminated with different concentrations of STEC. The STEC concentration in raw milk was checked by the microdilution method as described in Baron et al. (2006) with some modifications. We sampled 100 μL of each contaminated milk and first diluted it in 900 μL of PBS. After intense vortexing (3,000 rpm during 5 s), these aliquots were placed in a sterile 96-well microplate (Greiner) and 10-fold dilutions in PBS were performed. For each dilution, 20 μL was immediately dropped twice in a 24-well plate containing 1 mL of Luria Bertani (LB) agar. Once the drops were absorbed, the plates were incubated for 18–24 h at 37°C. The STEC concentration was estimated from wells containing 15–50 colonies. Raw milk suspensions were placed at 4°C for 16–24 h to allow natural cream separation (creaming). After creaming, the volume of the cream layer was evaluated by reading directly on the tube and STEC count in cream was evaluated by the microdilution method as previously described. The saturation concentration (SC) was evaluated by non-linear regression as previously described (Bagel et al., 2022a).

### 2.7. Impact of STEC gene deletions on capacity to adhere to MFGMPs

This experiment was performed as previously described (Bagel et al., 2022b). Briefly, 96-well plates (Nunc MaxiSorp plate, Thermo Fisher Scientific) were coated overnight at 20°C with 100 μL of MFGMP solutions at 100, 25, 6.25, 1.56, 0.39, or 0 μg/mL. After intensive washing, plates were blocked for 2 h at the same temperature with 250 μL of 5% Tween 20 phosphate buffer. The bacterial adhesion step was fixed for 2 h at 4°C in static conditions with 100 μL of PBS-washed STEC culture calibrated at 8 log_10_ CFU/mL. Plates were washed intensively to remove non-adherent bacteria. The number of adherent STEC cells was estimated by the growth delay. Bacterial growth was initiated with the addition of 100 μL of LB medium to each well. Plates were incubated in a plate reader (Spark, Tecan) inside a large humidity cassette at 37°C and the absorbance at 600 nm was measured every 5 min for 15 h. For each growth curve, a 5-parameter log-logistic fit was used to estimate the time (in seconds) to reach a 0.02 absorbance threshold (Tt; Time Threshold).

### 2.8. Data processing and statistical analysis

Data processing and statistical analysis were performed in R software (R Core Team, 2021). The normal distribution and homogeneity of the data and, if necessary, the residuals of the models, were checked graphically and statistical tests such as the Shapiro–Wilk, Levene, and Barlett tests were performed. Depending on the results of these tests, one-way ANOVA or Kruskal–Wallis tests were used to statistically evaluate the differences between groups. Multiple pairwise comparisons were realized with the Tukey method and Dunn’s test (value of *p*s were adjusted with Holm’s method) for parametric and non-parametric tests, respectively. For the natural creaming assay of STEC in competition with STEC surface proteins, the median effective dose (ED_50_) was estimated with the R “drc” package (Ritz et al., 2015). For this purpose, the concentration factor (CF) was log2-transformed and a dose–response model (log-logistic model at four parameters) was used. To evaluate the impact of gene KO on MFGMP adhesion, linear regressions with interaction terms for each type of strain were performed with the R “stats” package. To bypass the infinite value induced by log transformation, the concentration of the MFGMP solution (independent variable) was expressed as log(*X* + 1). For all statistical tests, a value of *p* ≤0.05 was considered significant. When necessary, the 95% confidence interval (CI) of the estimated model parameters was indicated in the following sections as follows: [lower bound; upper bound].

## 3. Results

### 3.1. Strain-specific inhibition of STEC concentration in the cream layer by STEC surface proteins

**Figure 1:**
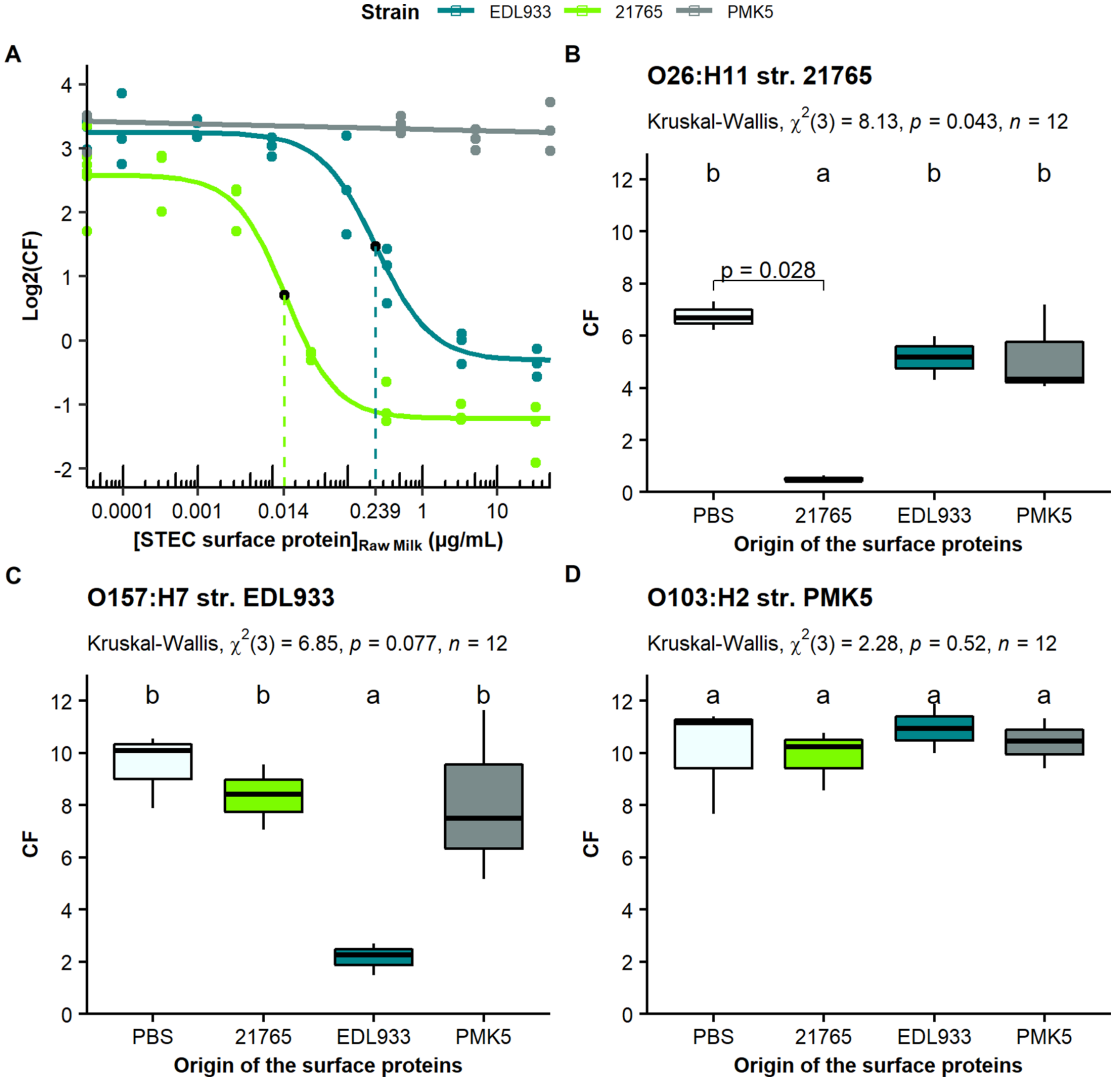
Natural creaming assay of STEC in competition with free STEC surface proteins. **(A)** Effect of STEC surface protein supplementation in raw milk (μg/mL) on the concentration factor of STEC in the cream layer (CF). Raw milk was incubated for 2 h at 4°C with increasing concentrations of STEC surface proteins from STEC str. 21765, str. EDL933, or str. PMK5. Then, raw milk was supplemented at 6 log_10_ CFU/mL with intact STEC cells corresponding to the same strain used for protein supplementation. CF of STEC was log_2_-transformed and the final concentration of STEC surface proteins in raw milk is represented in a log_10_ scale. Black points represent the median effective dose (ED_50_ in μg/mL) of each dose–response curve. The effect of the strain origin of surface proteins on the concentration of STEC str. 21765 **(B)**, EDL933 **(C)**, and PMK5 **(D)** in cream is presented on the corresponding panels. Raw milk was supplemented with STEC surface protein at a final concentration of 0.20 μg/mL and then inoculated individually at 6 log_10_ CFU/mL with each STEC strain. Both experiments were performed in triplicate with independent bacterial cultures (*n* = 3). The colors represent the different strains. The control condition (+PBS) is shown in light blue. Groups (a and b) were defined for *p* < 0.051 according to Tukey test pairwise comparisons.

To determine the involvement of STEC surface proteins in the STEC-MFG association, we developed an assay to evaluate the competition between free and membrane-anchored STEC surface proteins for STEC concentration in the cream layer. Addition of STEC surface proteins to raw milk decreased the concentration of STEC in the cream layer (Figure 1A). STEC concentration in cream as a function of added protein concentration displayed a dose–response type curve for O157:H7 str. EDL933 and O26:H11 str. 21765. For both strains, a distinct median effective dose (ED50) was estimated at 0.014 μg/mL with a 95% CI [0.01; 0.02] for O26:H11 str. 21765 and 0.24 μg/mL with a 95% CI [0.13; 0.35] for O157:H7 str. EDL933 (Table 2). The concentration factor (CF) of STEC in the cream layer was calculated using the inverse log2 function from the estimated parameters of dose–response models. When STEC surface proteins were not added (Table 2; parameter c), STEC cells were concentrated 6.24 fold with a 95% CI [5.12; 7.60] and 9.47 fold with a 95% CI [8.01; 11.21] in cream for O26:H11 str. 21765 and O157:H7 str. EDL933, respectively, compared with the initial concentration in raw milk (6 log_10_ CFU/mL). Although, with the highest concentration of surface proteins (Table 2; parameter d), the concentration of STEC in the cream was less accurate. O157:H7 str. EDL933 displayed a CF of 0.81 with a 95% CI [0.61; 1.07] and O26:H11 str. 21765 had a CF of 0.43 with a 95% CI [0.35; 0.52].

**Table 2:** Estimation of the coefficients of the log-logistic regressions (dose response) of the natural creaming assay of STEC in competition with free STEC surface proteins.

	O26:H11 str. 21765				O157:H7 str. EDL933			
	b	c	d	e	b	c	d	e
Estimate	1.23	−1.22	2.64	0.014	1.18	−0.31	3.25	0.24
CI95%	[0.63; 1.84]	[−1.515; −0.931]	[2.36; 2.93]	[0.01; 0.02]	[0.39; 1.97]	[−0.72; 0.09]	[3.00; 3.49]	[0.13; 0.35]
Std.	0.29	0.14	0.14	0.00	0.38	0.19	0.12	0.05
Error	4.28	−8.73	19.38	4.09	3.13	−1.60	27.95	4.66
t-value	0.00	0.00	0.00	0.00	0.01	0.13	<2.2e-16	0.00
value of *p*	^***^	^***^	^***^	^***^	^**^		^***^	^***^

b, the slope at the median effective dose (ED_50_); c, the lower limit of the curve; d, the upper limit of the curve; e, the median effective dose (ED_50_). ^***^Value of *p* ≤ 0.001 and ^**^Value of *p* ≤ 0.01.

Furthermore, inhibition of STEC concentration in cream was only observed when surface proteins of the same strain were initially added to raw milk (Figures 1A,B). A parametric test showed significant differences for O26:H11 str. 21765 and O157:H7 str. EDL933 (group ‘a’; Figures 1B,C). Due to the low number of data points, we also performed a non-parametric test, and a difference was only observed for O26:H11 str. 21765 between control (PBS) and when O26:H11 str. 21765 surface proteins were added (value of *p* = 0.028). In contrast, no inhibitory effects of the surface proteins from O103:H2 str. PMK5 were observed in the PMK5 strain itself (Figure 1A) or in the other two strains (Figure 1D).

### 3.2. STEC surface proteins associated with the MFGM

**Figure 2:**
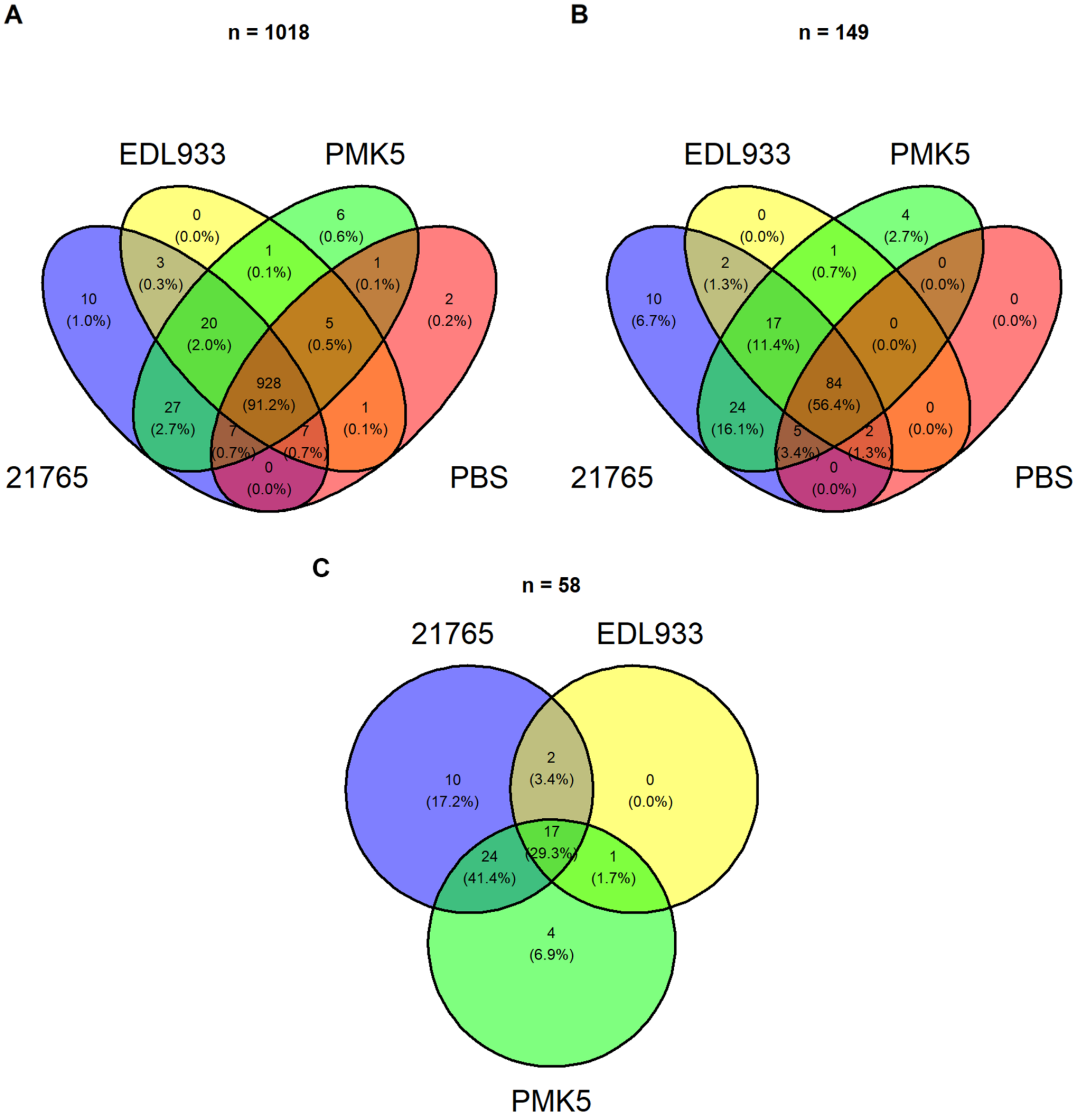
*Escherichia coli* proteins identified by MS/MS. Venn diagram illustrating **(A)** total and **(B)** *E. coli*-specific proteins identified in protein extracts of MFGMPs from raw milk enriched with STEC surface proteins (O26:H11 str. 21765, O157:H7 str. EDL933, or O103:H2 str. PMK5) or control (PBS). Sample-specific *E. coli* proteins enriched in STEC surface proteins are presented in panel **(C)**.

Next, we studied whether proteins from the STEC extract were responsible for the inhibition of intact STEC cells in the cream layer. We hypothesized that some of the extracted STEC surface proteins bind to MFGMs, thereby blocking access to binding sites for surface proteins anchored in the STEC membrane. We sought to identify these proteins and, to this end, we used mass spectrometry on the total protein extract from MFGM obtained after natural creaming of the milk enriched with STEC surface proteins. By mass spectrometry, 1,042 proteins were identified regardless of the sample (*n* = 12), including 869 proteins associated with *Bos taurus*, 149 proteins associated with *E. coli*, and 24 proteins from the contaminant library (Supplementary Table S2). Approximately 91.2% of identified proteins (928) were common to all samples (Figure 2A). Among the 149 proteins associated with *E. coli* (Figure 2B), only 58 proteins were specific to the samples supplemented with STEC protein extract (Figure 2C). A list of all proteins associated with *E. coli* is available in Supplementary Table S2. Among the 58 proteins specific to the STEC protein-supplemented samples, only 34 proteins were differentially expressed ≥2-fold between the strains (DEPs) (value of *p* ≥0.05): 21 DEPs were associated with O26:H11 str. 21765, 12 with O103:H3 str. PMK5, and 1 with O157:H7 str. EDL933 (Table 3). A large majority of the identified *E. coli* proteins were predicted to be cytoplasmic, including ribosomal proteins, transcription factors, and enzymes, while four unique proteins (OmpA, OmpN, FliC, and FadL) were related to surface localization (Table 3). Further experiments focused only on OmpA and FliC.

**Table 3:** Relative quantification of STEC proteins associated with the MFGM protein fraction.

Accession	Accession homologous	Protein names	PSM^*^	Localization	Absence/presence			Log2 Ratio (value of *p*)		
					21765	EDL933	PMK5	21765/PMK5	21765/EDL933	EDL933/PMK5
DEPs in O26:H11 str. 21765 (*n* = 21)										
A0A7B4KCG4	Q3Z601	Heat shock 70 kDa protein	113	Cytoplasmic	+	−	+	1,29 (4,50E−03)	6,64 (1,00E−17)	−6,64 (1,00E−17)
A0A4C9D1M4	−	50S ribosomal protein L7/L12	24	Cytoplasmic	+	−	+	1,54 (9,01E−04)	6,64 (1,00E−17)	−6,64 (1,00E−17)
A0A2T1LE41	−	Elongation factor G (EF-G)	11	Cytoplasmic	+	−	−	6,64 (1,00E−17)	6,64 (1,00E−17)	−
A0A783S9P7	B7NDU8	Elongation factor G (EF-G)	10	Cytoplasmic	+	−	+	1,31 (4,02E−03)	6,64 (1,00E−17)	−6,64 (1,00E−17)
A0A6M0PNN9	A9N0J3	50S ribosomal protein L7/L12	10	Cytoplasmic	+	−	−	6,64 (1,00E−17)	6,64 (1,00E−17)	−
Q1R7A8	−	Fructose-bisphosphate aldolase (FBP aldolase)	5	Cytoplasmic	+	−	+	1,32 (3,90E−03)	6,64 (1,00E−17)	−6,64 (1,00E−17)
A0A6M0PFC9	A0A1B7K6Y0	50S ribosomal protein L5	3	Cytoplasmic	+	−	+	1,23 (6,64E−03)	6,64 (1,00E−17)	−6,64 (1,00E−17)
A0A7I6R742	P0A9Q7	Bifunctional aldehyde-alcohol dehydrogenase AdhE	2	Cytoplasmic	+	−	−	6,64 (1,00E−17)	6,64 (1,00E−17)	−
Q8XEB8	−	Isochorismatase domain-containing protein	1	Cytoplasmic	+	+	−	6,64 (1,00E−17)	2,33 (1,00E−17)	6,64 (1,00E−17)
F4TL18	−	DNA-directed RNA polymerase subunit alpha	1	Cytoplasmic	+	+	+	1,65 (4,00E−04)	1,22 (3,82E−03)	0,43 (3,81E−01)
A0A377DM26	−	50S ribosomal protein L4	1	Cytoplasmic	+	+	+	1,81 (1,12E−04)	2,08 (1,45E−08)	-0,26 (9,89E−01)
A0A5C9AEQ4	−	Cysteine synthase (Fragment)	1	Cytoplasmic	+	−	−	6,64 (1,00E−17)	6,64 (1,00E−17)	−
A0A1M2TW09	−	Trigger factor (TF)	1	Cytoplasmic	+	−	−	6,64 (1,00E−17)	6,64 (1,00E−17)	−
A0A5P0ZBF6	A0A8F3FHV1	NAD(P)H dehydrogenase	1	Cytoplasmic	+	−	−	6,64 (1,00E−17)	6,64 (1,00E−17)	−
A0A7X1MK38	−	Dihydrolipoyl dehydrogenase	1	Cytoplasmic	+	−	+	1,56 (7,72E−04)	6,64 (1,00E−17)	−6,64 (1,00E−17)
A0A6M0PNA7	−	Transaldolase	1	Cytoplasmic	+	−	−	6,64 (1,00E−17)	6,64 (1,00E−17)	−
A0A4C9WKT1	−	Glucose−6-phosphate isomerase	1	Cytoplasmic	+	−	−	6,64 (1,00E−17)	6,64 (1,00E−17)	−
A0A0B1MZJ0	−	Protein ElaB	5	CytoplasmicMembrane	+	−	+	2,65 (3,66E−08)	6,64 (1,00E−17)	-6,64 (1,00E−17)
A0PCV7	A0PCV7	Flagellin (FliC)	1	Extracellular	+	−	−	6,64 (1,00E−17)	6,64 (1,00E−17)	−
A0A1X3J431	−	Outer membrane protein A (OmpA)	62	OuterMembrane	+	+	+	1,77 (1,52E−04)	6,63 (1,00E−17)	−4,86 (1,00E−17)
A0A0A0FCD2	−	Long-chain fatty acid transport protein (FadL)	1	OuterMembrane	+	−	−	6,64 (1,00E−17)	6,64 (1,00E−17)	−
DEPs in O103:H3 str. PMK5 (*n* = 12)										
A0A3Y1QZA3	−	Chaperonin GroEL	63	Cytoplasmic	+	+	+	−1,16 (4,69E−02)	0,57 (5,02E−03)	−4,02 (1,00E−17)
A0A140NF01	−	Transcription termination factor Rho	6	Cytoplasmic	+	+	+	−2,59 (1,46E−06)	−0,54 (1,52E−01)	−2,15 (1,05E−02)
A0A6M0PDM3	A0A1B7JZZ4	Ribosome-binding ATPase YchF	5	Cytoplasmic	+	−	+	−1,84 (8,74E−04)	6,64 (1,00E−17)	−6,64 (1,00E−17)
A0A6N7NAY3	A0A059UV59	50S ribosomal protein L15	5	Cytoplasmic	+	+	+	−3,11 (5,33E−09)	0,09 (7,98E−01)	−3,4 (3,71E−09)
A0A6M0Q291	−	Uracil phosphoribosyltransferase	4	Cytoplasmic	+	−	+	−2,14 (8,53E−05)	6,64 (1,00E−17)	−6,64 (1,00E−17)
A0A6N7NHK1	Q1IFW5	50S ribosomal protein L4	4	Cytoplasmic	+	+	+	−2,53 (2,65E−06)	−0,28 (4,29E−01)	−2,45 (3,14E−04)
D6I1F1	A0A376TSQ8	Translation initiation factor IF−2	3	Cytoplasmic	+	+	+	−2,03 (2,13E−04)	−0,22 (5,38E−01)	−2,32 (5,47E−03)
A0A6N7NGA0	−	Aspartyl/glutamyl-tRNA(Asn/Gln) amidotransferase	1	Cytoplasmic	−	−	+	−6,64 (1,00E−17)	0,00E+00	−6,64 (1,00E−17)
A0A6D1A8Y1	−	F0F1 ATP synthase subunit beta (Fragment)	5	CytoplasmicMembrane	−	−	+	−6,64 (1,00E−17)	−	−6,64 (1,00E−17)
F4NDT5	−	Outer membrane protein N	33	OuterMembrane	−	+	+	−6,64 (1,00E−17)	−6,64 (1,00E−17)	−3,68 (3,85E−11)
A0A1X3J1X3	−	Bacteriophage Mu tail sheath protein (GpL)	3	Other (Phage)	−	−	+	−6,64 (1,00E−17)	−	−6,64 (1,00E−17)
A0A2X9QJ57	−	Head protein (Putative phage major head subunit)	1	Other (Phage)	−	−	+	−6,64 (1,00E−17)	−	−6,64 (1,00E−17)
DEPs in O157:H7 str. EDL933 (*n* = 1)										
A0A4T8WWM6	A0A0H3JH81	3-oxoacyl-[acyl-carrier-protein] synthase I	1	Cytoplasmic	+	+	−	6,64 (1,00E−17)	−2,25 (1,00E−17)	6,64 (1,00E−17)

^*^PSM, peptide spectrum matches. A protein was considered present if a unique peptide was detected in at least one of the triplicate samples. Relative quantification, indicated by the log2 ratio and associated value of p (*t*-test), refined the comparison between strains by showing their difference in expression. A homologous protein (>90%) was used to predict function and subcellular localization if necessary. +, presence of the protein. -, absence of the protein.

### 3.3. Impact of STEC gene knockout on the saturation of the cream layer by natural creaming

**Figure 3:**
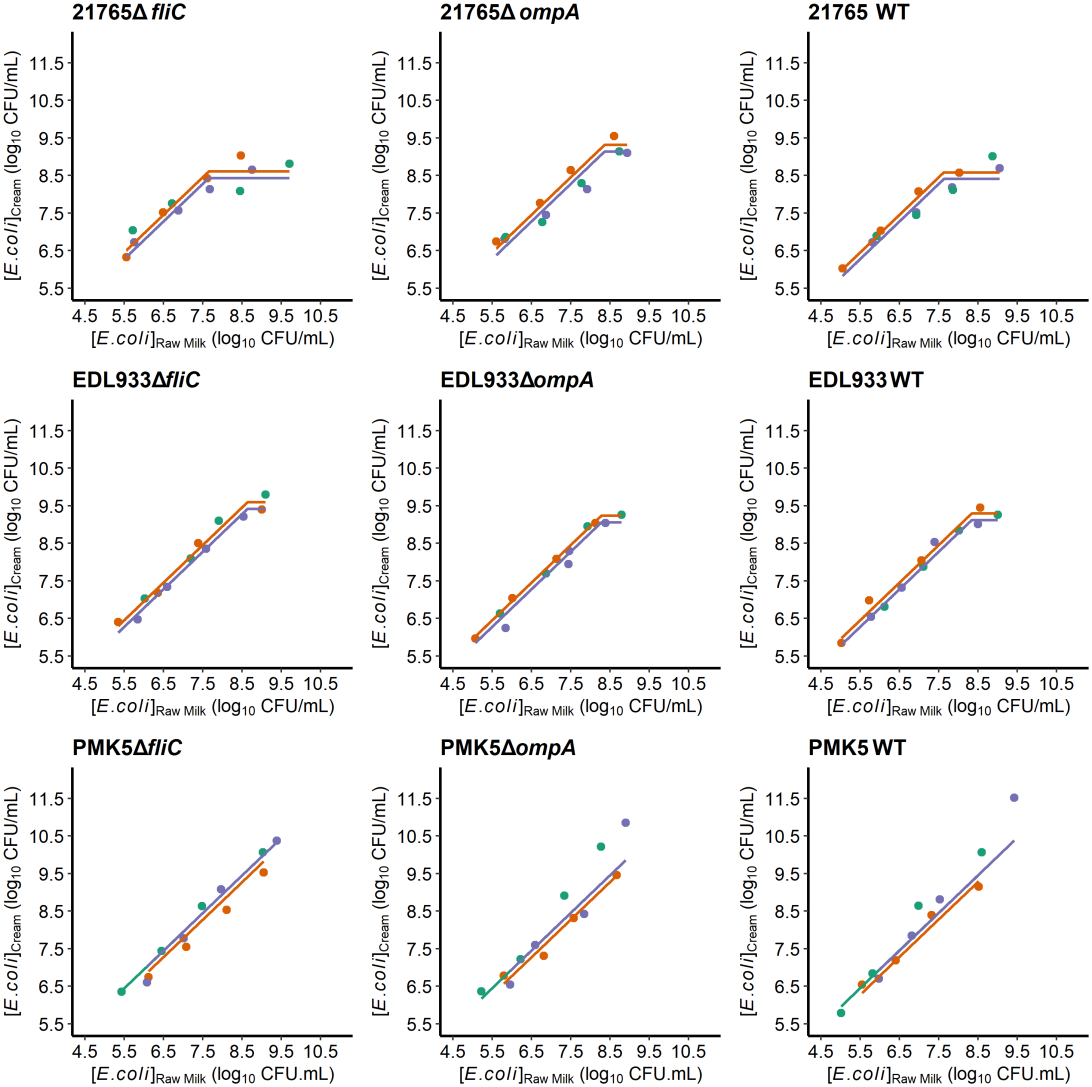
Saturation curves of STEC strains and their derived mutants (Δ*fliC* and Δ*ompA*) in the raw milk cream layer obtained by natural creaming assay. The concentration of each strain in the raw milk cream layer as a function of the initial concentration in milk was plotted for each milk used (points) as well as the regression curve (flat line). Colors represent different milks (*n* = 3). Bovine raw milks were purchased either from the local market or directly from farms. They were purchased over a short period of time. This means that they may come from different animals, different milking and could have microbiological composition. For two milks, the coefficient of the linear portion of the regression curve was the same value (same volume of cream). Therefore, these curves are superimposed on the graphs.

**Figure 4:**
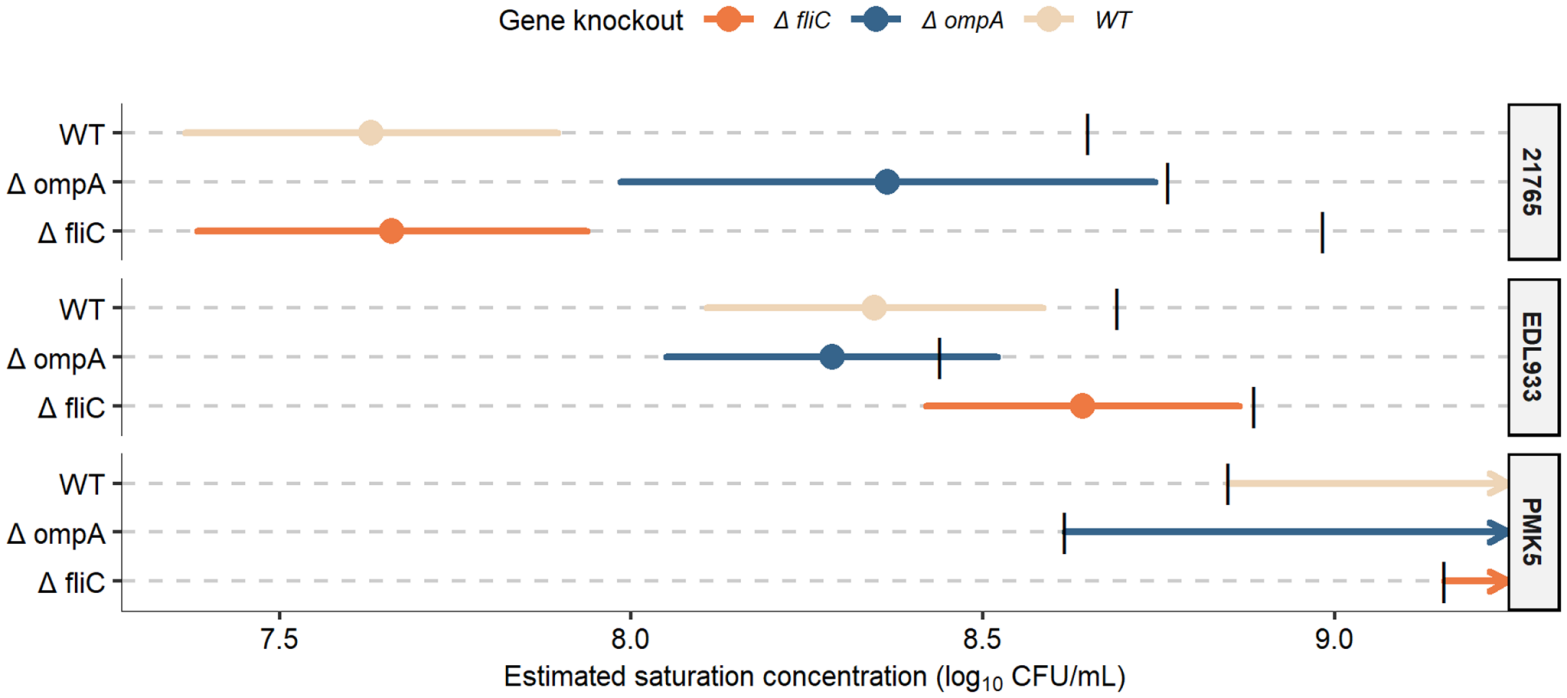
Estimation of the saturation concentration (SC) of STEC strains and their derived mutants (Δ*fliC* and Δ*ompA*) in the raw cream layer from natural creaming assays. Segments represent the 95% confidence intervals (CI). For O103:H2 str. PMK5 WT, Δ*fliC*, and Δ*ompA*, the model could not estimate an SC, which was considered as non-saturation in these conditions. Black bars represent the mean of the maximal spiked level (Cmilk_max_). Strains with an SC above this threshold, such as O157:H7 str. EDL933 Δ*ompA*, were considered non-saturating under these conditions. Each knockout gene is displayed by a distinct color.

To confirm the potential role of the two candidate STEC receptors for MFG binding, namely the FliC and OmpA proteins, we used a mutagenesis approach coupled with phenotype observation to assess their ability to adhere to MFG proteins and concentrate in the cream layer. The concentration of STEC and their derived mutants in the cream layer was ~1 log_10_ CFU/mL greater than the initial enrichment level (Figure 3). STEC strains were mainly recovered in the cream layer when the bacterial level in raw milk ranged from 5 to 7.5 log_10_ CFU/mL or from 5 to 8.5 log_10_ CFU/mL, depending on the strain (Figure 3). However, at higher inoculum levels (7.5 or 8.5 log_10_ CFU/mL), the concentration of *E. coli* in the cream was limited; the cream layer appeared saturated by STEC. The estimated SC were between 7.63 and 8.64 log_10_ CFU/mL (min; max) (Figure 4). Deletion of the *ompA* gene significantly altered the concentration of STEC in the cream for O26:H11 str. 21765. The SC of O26:H11 str. 21765 Δ*ompA* was estimated at 8.36 log_10_ CFU/mL with a 95% CI [7.98; 8.74], while the WT strain (control) reached saturation of the cream layer at a concentration of 7.63 log_10_ CFU/mL with a 95% CI [7.37; 7.90] (Figure 3). In contrast, O26:H11 str. 21765 Δ*fliC* presented a profile similar to the WT strain, saturating the cream layer at an SC estimated at 7.66 with a 95% CI [7.38; 7.93]. The derived strains from O157:H7 EDL933 displayed no significant difference in SC compared with the WT strain. All SC were estimated between 8.05 and 8.86 (the min and the max of lower and upper bounds of the 95% CI). For the strains associated with O103:H2 str. PMK5, the model could not estimate the SC due to the lack of a point in the saturation phase, which would result in an SC higher than the highest concentration assayed.

### 3.4. Impact of STEC gene knockout on capacity to adhere to MFGMPs

**Figure 5:**
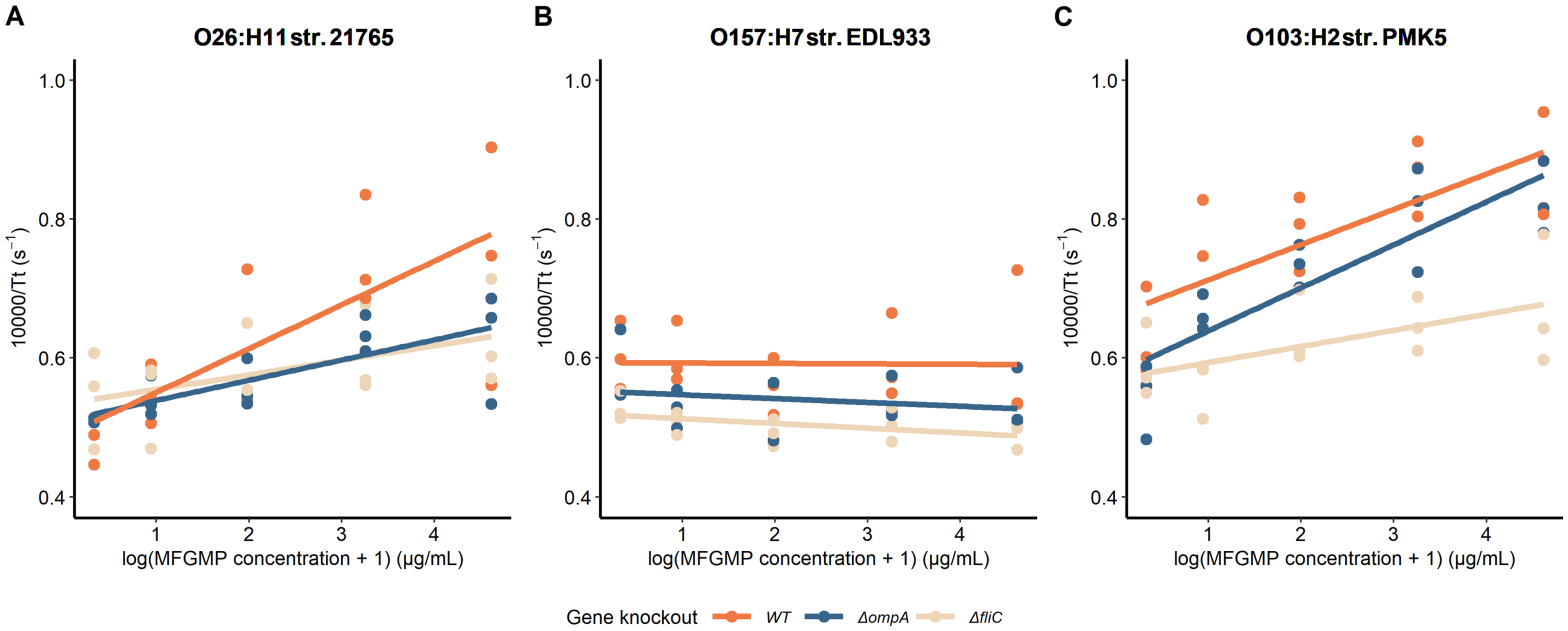
Capacity of wild-type strains **(A)** O26:H11 21765, **(B)** 0157:H7 EDL993, and **(C)** O103:H2 PMK5 and derived mutants to adhere to milk fat globule membrane proteins (MFGMPs). A plate adhesion assay was performed and the number of adherent STEC cells was estimated by the growth time delay (Tt) according to a threshold of the OD_600_ at 0.02. A shorter Tt meant a larger number of cells, while a longer time meant a larger number of adherent cells. The Tt was represented by its inverse to facilitate interpretation and linear regressions were plotted to visualize the effect of protein quantity on adhesion. Each knockout gene is shown in a different color.

The *fliC* and *ompA* knockouts appeared to decrease the adhesion to MFGMPs of the three WT strains assayed (Figure 5). To confirm this observation, slope comparisons between mutants and WT strains were made by linear regression with interaction terms (Table 4). Estimation of the coefficient of slopes statistically confirmed the graphical observations only for the fliC knockouts of O26:H11 str. 21765 (value of *p* = 0.031) and O103:H2 str. PMK5 (value of *p* = 0.034) (interaction term; Table 4). For O157:H7 str. EDL933 and its derived mutants, no difference in the slope coefficient was observed. Moreover, the slope coefficient of the WT strain was not statistically different from zero. Therefore, the assayed O157 strains showed little or no affinity for MFGMPs. The basal adhesion (absence of MFGMPs) of mutated strains was not significantly different to that of WT strains, except for O157:H7 str. EDL933ΔfliC (Table 4).

**Table 4:** The outputs of linear regression of the STEC-MFGMP plate adhesion assays.

	O26:H11 str. 27,165	O103:H2 str. PMK5	O157:H7 str. EDL933
(Intercept)	0.488^***^ [0.429, 0.548]	0.616^***^ [0.563, 0.669]	0.584^***^ [0.552, 0.616]
	(0.030)	(0.026)	(0.016)
log(MFGMPs+1)	0.058^***^ [0.034, 0.082]	0.065^***^ [0.043, 0.086]	0.002[−0.011, 0.015]
	(0.012)	(0.011)	(0.006)
genotypefliC	0.048 [−0.036, 0.132]	−0.074 [−0.149, 0.002]	−0.076^**^ [−0.121, −0.031]
	(0.042)	(0.037)	(0.022)
genotypeompA	0.019 [−0.065, 0.103]	−0.066 [−0.141, 0.010]	−0.043 [−0.088, 0.002]
	(0.042)	(0.037)	(0.022)
log(MFGMPs+1) × genotypefliC	−0.037^*^ [−0.071, −0.004]	−0.033^*^ [−0.063, −0.003]	−0.006 [−0.024, 0.013]
	(0.017)	(0.015)	(0.009)
log(MFGMPs+1) × genotypeompA	−0.028 [−0.062, 0.006]	0.005 [−0.025, 0.036]	−0.004 [−0.022, 0.014]
	(0.017)	(0.015)	(0.009)
Num. Obs.	54	54	54
*R* ^2^	0.415	0.711	0.419
*R*^2^ Adj.	0.354	0.681	0.358
RMSE	0.08	0.07	0.04

Regressions were performed separately for each strain and the wild-type (WT) strain was used as a reference group to compare the coefficients with its derived mutants. The inverse of the time threshold (10,000/Tt in sec^−1^) was used as a response variable. The 95% confidence intervals are indicated in square brackets and the standard errors are in parentheses. ^***^Value of *p* < 0.001, ^**^Value of *p* < 0.01, and ^*^Value of *p* < 0.05.

## 4. Discussion

This study investigated the role and specificity of STEC surface proteins in the mechanism of STEC-MFGM association by natural creaming assay and plate adhesion assay.

We found that supplementation of raw milk with STEC surface proteins decreased STEC concentration in cream in a dose–response manner for O157:H7 str. EDL933 and O26:H11 str. 21765 (Figure 1A). The estimated median effective dose (ED_50_) was almost 20 times higher for strain O157 than for strain O26suggesting different proportions of bioactive proteins in the extracts of each strain. Without the addition of surface proteins, O157:H7 str. EDL933 was approximately 2 times more concentrated in the cream layer than O26:H11 str. 21765. This may explain, in part, the greater amount of protein required to observe an effect. In addition, we know that O26:H11 str. 21765 saturates more rapidly than O157:H7 str. EDL933. Thus, according to our hypothesis, STEC strain 21765 would recognize fewer binding sites or perhaps different affinity with the same number of binding sites on the surface of MFGs than EDL933, and therefore fewer proteins would be needed to block its concentration in the cream layer.

The lack of inhibitory effect observed for O103:H3 str. PMK5 (Figures 1A,D) could be explained in part by our experimental methods. First, the extraction yield can be different between strains. STEC strains are known to present a large genetic diversity (Douëllou et al., 2016, 2017a). Thus, depending on the type of proteins expressed by each strain, it may be more or less difficult to extract the proteins that are highly anchored in the membrane. Second, protein conformation after extraction may be different from native conformation, hiding epitopes involved in adhesion. Third, the affinity of O103:H3 str. PMK5 may be based on a more complex and multifactorial mechanism than other strains. In addition, we set the membrane protein adhesion conditions to 2 h at 4°C to be consistent between experiments. While we previously showed that this time was sufficient to allow STEC adhesion to MFGMPs (Bagel et al., 2022b), a longer time may override the lack of effect observed with O103:H3 str. PMK5.

Our crossing-competition assays showed a lack of complementarity with protein extracts from other STEC strains. It would suggest that the inhibition of this pathogen in the cream layer is potentially related to a strain-specific mechanism. To the authors’ knowledge, no similar study, even on other bacterial species, has been published. These studies are the first to be performed with STEC.

The inhibition associated with free STEC membrane proteins seems to be more related to a repulsion effect of STEC cells from the cream layer than to an antimicrobial effect. Surface protein extracts did not affect the viability of the strains after 24 h at 4°C (data not shown). In addition, if there was an antimicrobial effect, the concentration of STEC should be lower at high concentrations of added proteins.

The addition of external elements, can disrupt the balance of ionic and electrical forces native and induce aggregation of MFGs or milk proteins (caseins, whey proteins) and block STEC access to adhesion sites. Given the results obtained in the crossing experiment, we believe that the addition of protein only slightly modified the stability of milk. The use of potentially highly pathogenic bacterial strains limits access to other techniques and instruments that could identify a change in the physical stability of the MFGs (fat globule size, aggregation) and their surface charge (zeta potential). Raw milk also contains a rich microbiota that could bias our results by adsorbing on the surface of MFGs. However, as explained in an exploratory study, the concentration of microflora compared with the experimental concentration of STEC, is negligible (Bagel et al., 2022a).

Due to the small sample size, the power of statistical tests was quite low, although significant effects were demonstrated. Data checked all conditions for a parametric test, but as a precaution, we also performed a non-parametric test. Further studies with more replicates are needed to confirm the specificity of surface protein extracts in the inhibition of bacterial adhesion. Furthermore, the biological question could be broadened by studying whether the inhibition effect is conserved within a serotype.

Thus, a label-free proteomic study was performed to identify the STEC surface proteins associated with the MFGM fraction after natural creaming.

The major MFGMPs, such as xanthine dehydrogenase/oxidase, butyrophilin, and lactadherin (Ortega-Anaya et al., 2022) were identified in all samples. In addition, no proteins from the aqueous phase of raw milk such as casein or β-lactoglobulin were identified. These data confirm that the method used for MFGMP extraction was effective and therefore, the STEC proteins identified are likely to be strongly associated with the MFGM protein fraction. Among the identified STEC membrane-associated proteins, the Outer membrane protein A (OmpA) and the flagellin (FliC) warranted further investigation (explained below). Therefore, we created mutant strains to assess the involvement of these proteins in the concentration of STEC strains in cream and their ability to adhere to MFGMPs.

The protein OmpA, a well-known bacterial protein involved in adhesion (Hirakawa et al., 2021), was identified in all samples but was found in significantly higher levels in samples supplemented with surface proteins derived from O26:H11 str. 21765. A previous study showed that protein extracts of the three strains studied here had no significant difference in OmpA expression (Bagel et al., 2022b), suggesting a preferential affinity of the OmpA variant of O26:H11 str. 21765. In this study, *OmpA* deletion resulted in a significant higher concentration of the O26:H11 str. 21765 mutant compared with the WT strain only for, but not for other strains. The effect of *ompA* deletion of strain O26 is the opposite of what we expected. This phenomenon is probably related to mechanisms other than adhesion to MFGMPs since all three mutants for *ompA* showed similar MFGMP adhesion abilities to the WT strains (Figure 5).

Alignment of the OmpA protein sequence showed an E → K mutation at residue 89 in O26:H11 str. 21765 that was not present in other strains (Supplementary Figure S1). This mutation provides partial susceptibility to phage M1 and increases resistance to colicin L (Morona et al., 1984, 1985). In addition, OmpA is a major target of mammalian host cell defense (Wang, 2002) and may be targeted by immune cells contained in raw milk. Therefore, *ompA* deletion could improve survival of this strain. Moreover, *ompA* knockout (KO) affects the shape and size of various bacteria, including the EDL933 strain, leading to compromised outer membrane integrity and spherical morphology (Nagy et al., 2015). Our results suggest that OmpA may be more involved in physiological interactions than in adhesive interactions.

The flagellin (FliC) was identified only in samples supplemented with surface proteins from O26:H11 str. 21765 (Supplementary Table S2). Regardless of the sample, only one peptide (FDSAITNLGNTVNNLSSAR) related to the FliC protein sequence was identified by MS/MS. This peptide is present in the FliC protein sequences of O26:H11 str. 21765 and O103:H2 str. PMK5 (data not shown). Interestingly, in our previous study, FliC protein was relatively more present in the protein extracts of other strains than in O26:H11 str. 21765 (Bagel et al., 2022b). We also found that the STEC-MFG association is not always stable and that bacteria can move around and between MFGs, suggesting the involvement of bacterial mobility in the affinity of STEC for MFGs (data not shown). In addition, given that flagellin has been widely described as a potential adhesin (Nedeljković et al., 2021) and is intrinsically linked to serotype, we speculated that it may play a key role in the STEC-MFGM association. Nevertheless, the flagellin FliC did not seem to be strongly involved in the concentration of STEC in the cream (Figures 3, 4).

Although *fliC* deletion did not decrease the concentration of STEC in cream, a decrease in the attachment to MFGMPs was noted (Figure 5). The effect seemed to be more pronounced for O103:H2 str. PMK5 than the other two strains. Interestingly, in experimental culture conditions used for raw milk spiking (BHI; 37°C), O26:H11 str. 21765 did not show mobility in a low agar content plate, unlike O157:H7 str. EDL933 and O103:H2 str. PMK5 (data not shown). The lack of motility may reflect the low *fliC* expression in O26:H11 str. 21765 and therefore, this protein may not be involved in STEC-MFG association in this strain. These differences could explain the stronger inhibition of adhesion seen in O103:H2 str. PMK5Δ*fliC*, compared with O26:H11 str. 21765Δ*fliC*. The motility of STEC in raw milk and/or their attraction to MFGs did not seem to be strongly involved in STEC concentration in the raw cream layer. This variation should be further studied in raw milk conditions.

Also, it is important to note that MFGMPs are in different physicochemical conditions and conformations in the plate adhesion and natural creaming assays. In natural creaming, the proteins are anchored in the MFGM and have a native conformation whereas, in plate adhesion assays, proteins have been extracted from their membrane. Therefore, their hydrophobic domain, which is normally anchored in the membrane, becomes available to bacteria. We do not know the conformation of these proteins under these conditions. Moreover, the plate adhesion assay was designed to assess the relative quantification of STEC adhesion at the population level. The resolution of the quantifying method may not be sufficient to observe a slight difference. In addition, we found that the relationship between the concentration of MFGMPs and the Tt was not strictly linear, especially for O103:H2 str. PMK5 (Figure 5). It appears that the strain reached saturation of its adhesion capacity or steric interference occurred at high concentrations of MFGMPs. However, the use of linear regression on these data allowed us to highlight a trend of adhesion profiles towards MFGMPs.

In addition to the OmpA and FliC proteins, we identified two other STEC membrane-associated proteins. The long-chain fatty acid transport protein (FadL) was identified in only one of the samples enriched with proteins from O26:H11 str. 21765. The outer membrane protein N (OmpN) was identified in samples enriched with protein extracts from O157:H7 str. EDL933 and O103:H2 str. PMK5. However, the peptide used for relative quantification was not specific to our strains. Raw milk is a non-sterile product and contains a rich and diverse microbiota, making it difficult to discriminate between added STEC proteins and native bacterial proteins in milk (O’Sullivan and Cotter, 2017). This was also the case for the outer membrane protein C (OmpC), another outer membrane protein involved in bacterial adhesion (Rolhion et al., 2007; Hejair et al., 2017) that was identified in all samples.

Additionally, a significant number of bacterial cytoplasmic proteins were also identified, suggesting a potential role in the inhibition of STEC concentration in the cream layer. Moreover, the real number of *E. coli* proteins associated with the protein fraction of MFGM is probably much higher than the number of proteins that we have identified by MS/MS, due to the complexity of the samples and the high proportion of MFGMPs compared to *E. coli* proteins. The addition of a decomplexation step for proteomic analysis showed an enrichment of more than 50% of bovine proteins compared with two additional proteins potentially associated with *E. coli* (data not shown). Furthermore, the addition of a decomplexation step was not sufficient to increase the number of proteins associated with *E. coli*. In addition, other STEC proteins may play a key role in association with MFGs, but if these proteins have low affinity and interaction strength for MFGMPs, they were most likely removed during the washing step and therefore could not be identified.

This work focused on the cellular and protein adhesion of STEC to the surface of raw MFGs or MFGMs under conditions close to those of raw milk (pH 6.8), but different from those of the human gut. The adhesion of STEC to the colon takes place at a neutral pH, similar to the pH of raw milk (Nugent et al., 2001). However, during digestion, MFGs pass through an acidic gastric phase that changes the MFGs and their surface properties. Therefore, the MFGs and their surface components in the gut are likely to be structurally different to those studied in our assays.

This study did not fully elucidate the STEC-MFG association, but it showed a complex underlying mechanism Better characterization of the STEC protein extract as well as development of an alternative holistic method could create new opportunities to understand the molecular interactions involved in STEC-MFG association. Further studies are also needed to explore the genetic regulation of STEC in raw milk supplemented with surface proteins. The association of bacteria, including STEC, to MFGs, is likely to be a complex and multifactorial event.

## Data availability statement

The datasets presented in this study can be found in online repositories. The names of the repository/repositories and accession number(s) can be found in the article/Supplementary material.

## Author contributions

AB: conceptualization, methodology, investigation, formal analysis, visualization, and writing—original draft. MB-C, MC, BH, and LC: investigation. CL: resources and writing— review and editing. VM: writing—review and editing. TD: writing—review and editing and funding acquisition. DS: conceptualization, writing—original draft, funding acquisition, and project administration. All authors contributed to the article and approved the submitted version.

## Funding

This work was supported by the Centre National Interprofessionnel de l’Economie Laitière (CNIEL, French Dairy Interbranch Organization, Paris, France), the French Ministry of Agriculture (Paris, France), and VetAgro Sup (Marcy-l’Etoile, France).

## Conflict of interest

The authors declare that the research was conducted in the absence of any commercial or financial relationships that could be construed as a potential conflict of interest.

## Publisher’s note

All claims expressed in this article are solely those of the authors and do not necessarily represent those of their affiliated organizations, or those of the publisher, the editors and the reviewers. Any product that may be evaluated in this article, or claim that may be made by its manufacturer, is not guaranteed or endorsed by the publisher.
