# Supplementary material for: Surface proteins of Shiga toxin-producing Escherichia coli mediate association with milk fat globules in raw milk
Source: Front Microbiol. 2023 Jun 23;14:1156374. doi: 10.3389/fmicb.2023.1156374 (PMC10328742; doi:10.3389/fmicb.2023.1156374)
Supplement: Supplementary file 3 [file Data_Sheet_1.docx]

Supplementary Material

**Surface proteins of Shiga toxin-producing Escherichia coli mediate association with milk fat globules in raw milk**

**A. Bagel^1^, M. Bouvier-Crozier^1, 2^, M. Canizares^2^, B. Hamadou^2^, L. Courcol^1^, C. Lopez^3^, V. Michel^4^, T. Douellou^1^ and D. Sergentet*^1, 2^**

*** Correspondence:**Pr. Delphine Sergentet, [delphine.sergentet@vetagro-sup.fr](mailto:delphine.sergentet@vetagro-sup.fr), +33478872599, 1 Avenue Bourgelat, VetAgro Sup, Marcy-L’Etoile, 69280, FRANCE

# Supplementary Figures and Tables

## Supplementary Figures

**
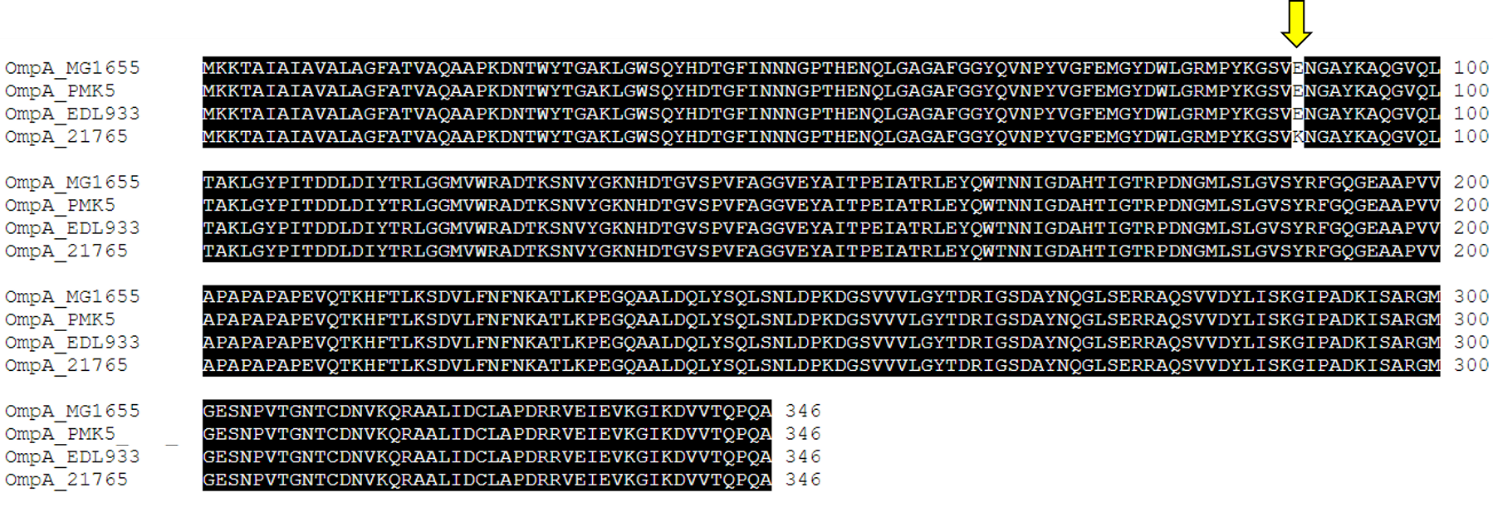
**

**Supplementary Figure 1.** Alignment of the OmpA sequences of the three STEC strains studied (O157:H7 EDL933, O103:H3 PMK5, and O26:H11 21765) and the non-pathogenic E. coli strain MG1655 K-12. An E → K mutation at residue 89 H11 was identified for O26:H11 str. 21765.

## Supplementary Tables

Supplementary Table 1. Primers and PCR conditions used for bacterial mutant construction.

Supplementary Table 2. Data of label-free relative quantification by mass spectrometry of MFGM-associated proteins in raw bovine milk after natural creaming. The first sheet groups all identified proteins. The second sheet summarizes only the proteins associated with *Escherichia coli* species.
